# Vasculitis distribution and clinical characteristics in giant cell arteritis: a retrospective study using the new 2022 ACR/EULAR classification criteria

**DOI:** 10.3389/fmed.2023.1286601

**Published:** 2023-11-13

**Authors:** Peter M. Andel, Andreas P. Diamantopoulos, Geirmund Myklebust, Glenn Haugeberg

**Affiliations:** ^1^Division of Rheumatology, Department of Rheumatology, Surgery, Inflammation and Transplantation, Oslo University Hospital Rikshospitalet, Oslo, Norway; ^2^Faculty of Medicine, Institute of Clinical Medicine, University of Oslo, Oslo, Norway; ^3^Research Department, Hospital of South East Norway, Kalnes, Norway; ^4^Division of Internal Medicine, Department of Infectious Diseases, Akershus University Hospital, Lørenskog, Norway; ^5^Research Department, Hospital of Southern Norway, Kristiansand, Norway; ^6^Division of Rheumatology, Department of Internal Medicine, Hospital of Southern Norway, Kristiansand, Norway; ^7^Department of Neuromedicine and Movement Science, Faculty of Medicine and Health Sciences, Norwegian University of Science and Technology (NTNU), Trondheim, Norway

**Keywords:** large-vessel vasculitis (LVV), giant cell arteritis (GCA), ultrasound, classification criteria, imaging

## Abstract

**Introduction:**

Giant cell arteritis (GCA) is the most common vasculitis of the elderly. In recent years, advanced imaging has to a certain extent replaced temporal artery biopsy (TAB) to aid diagnosis in many institutions and helped to identify three major phenotypes of GCA, namely, cranial GCA (c-GCA), large-vessel non-cranial GCA (LV-GCA), and a combination of these two patterns called mixed-GCA, which all show different clinical patterns. Recent 2022 American College of Rheumatology (ACR)/European League Against Rheumatism (EULAR) classification criteria respect the changing conception and clinical practice during the last two decades. In this cohort study, we present vasculitis distribution and baseline characteristics using the 2022 ACR/EULAR classification criteria as well as the EULAR core data set.

**Methods:**

In this retrospective study from Southern Norway, we identified all patients diagnosed with GCA between 2006 and 2019 in our single-center fast-track clinic (FTC). We included all patients who were examined using ultrasound (US) of cranial as well as non-cranial large vessels at diagnosis to depict vascular distribution. EULAR core data set, ACR 1990, and 2022 ACR/EULAR classification criteria were used to characterize the cohort.

**Results:**

Seventy-seven patients were diagnosed with GCA at our institution in the aforementioned period. Seventy-one patients (92.2%) were diagnosed with the help of US and included in the further analysis. The 2022 ACR/EULAR classification criteria allocated 69 patients (97.2%), while the ACR 1990 classification criteria allocated 49 patients (69.0%) in our cohort as having GCA. Mixed-GCA was the most common type in 33 patients (46.5%). Weight loss was significantly more common in patients with large-vessel non-cranial vasculitis in LV-GCA and mixed-GCA. Headache, on the other hand, was significantly more common in patients with involvement of cranial vessels.

**Conclusion:**

Mixed GCA was the most common form of GCA in our cohort. In our study, the 2022 ACR/EULAR classification criteria seem to be a more useful tool compared with the old ACR 1990 classification criteria to allocate GCA patients diagnosed and treated at our US-based FTC as having GCA.

## Introduction

Giant cell arteritis (GCA) is the most common form of large-vessel vasculitis in the elderly population (1). If left untreated, it poses a medical emergency due to impending vision loss and stroke risk (2). In certain subpopulations, GCA has also been associated with increased mortality (3, 4). GCA predominates in women and populations of northern European descent (5).

In the last two decades, advanced imaging techniques have changed the understanding of GCA, which seems to be a systemic, rather than a localized vasculitis of cranial arteries (2, 6, 7). Recent studies using positron emission tomography of radioactively labeled glucose (PET) or ultrasound (US) with experienced examiners and extended US protocols identified high rates of large-vessel involvement in GCA (6–9). These findings seem important as they were associated with refractory disease and specific complications such as posterior stroke in vertebral vasculitis or thoracic aortic aneurysm in aortitis (10–14). New classification criteria incorporating these new imaging modalities have recently been published by the American College of Rheumatology (ACR) together with the European Alliance Of Associations For Rheumatology (EULAR) and proved to be applicable to GCA cohorts (15, 16).

The fast-track clinic (FTC) approach incorporating US enables diagnosis and treatment within 48 h and has shown success in reducing vision loss (17, 18). Furthermore, outcome has been improved by new treatment options beyond prednisolone (19–21).

Southern Norway has consistently reported an annual incident rate among the highest in the world, though it shows a declining trend (4). US-based diagnosis was introduced in our rheumatology center on a regular basis in 2010. It has replaced temporal artery biopsy as the first diagnostic modality in diagnosing GCA while US-based FTC algorithms were finally implemented routinely in 2012 (18).

The primary aim of this study was to describe vasculitis distribution in cranial and non-cranial arteries in an FTC using US for diagnosis of GCA. Furthermore, we wanted to characterize our cohort using the 2018 EULAR core data set, the ACR 1990 classification criteria, and the new 2022 ACR/EULAR 2022 classification criteria for GCA (15, 22, 23).

## Method

All patients diagnosed with GCA at the central referral FTC in Agder County, Southern Norway, between 2006 and 2019 were retrospectively identified using the International Classification of Disease version 10 (ICD-10) coding system with the codes M31.5 and M31.6 in the central electronic hospital database.

All applicable medical records were thoroughly reviewed manually before the diagnosis was confirmed or rejected based on medical record information. Patients with a sustained diagnosis of GCA on the basis of clinics, imaging results, and temporal artery biopsy (TAB) were identified. Patients without US examinations at diagnosis were excluded for further analysis.

Data were collected in accordance with a structured protocol following the 2018 EULAR recommendations for a core data set to support observational research and clinical care in GCA. However, general disease assessment of patients and examiners was not routinely recorded in most patients prior to 2018 and was therefore not included, while history of cancer was not further stratified (22).

Standard US procedure contained an assessment of both temporal arteries (superficial temporal artery with frontal and parietal branches) in longitudinal and transversal planes with and without color Doppler mode. A positive US test was defined in the presence of hypoechoic vessel wall thickening (halo sign) that was confirmed by the compression sign (24, 25). The axillary and subclavian arteries were assessed in B-mode, and intima–media thickness (IMT) was measured in a longitudinal visualization. A positive test was defined if IMT > 1 mm (2). Other arteries, such as facial-, carotid-, and occipital arteries, were only sporadically assessed and therefore not further analyzed. The US examination was carried out at the FTC, 48 h after referral at the latest. US procedures were conducted by three experienced sonographers (APD, HB, and PMA) using Esaote (Esaote, Genua, Italy) machines up to 2019 and General Electric (General Electric Healthcare, Horten, Norway) Vivid machines in 2018 and 2019. Linear transducers were used with pre-specified settings according to common recommendations (26). Magnetic resonance imaging and PET were not part of a standard assessment and were only used sporadically. TAB was performed by the surgical department at the same hospital, and the specimens were assessed by several local pathologists.

Descriptive statistics were used to characterize the study cohort. Mean and standard deviation were calculated for continuous metric variables and frequencies for nominal and categorical variables.

To compare characteristics between the three major patterns of GCA, the chi-square test was used for categorical variables, and ANOVA and Bonferroni as a *post-hoc* test for continuous variables. Additionally, a multivariate analysis with multiple comparisons was conducted.

The level of significance of all tests was set at a *p*-value of ≤ 0.05. The Statistical Package for the Social Sciences (SPSS), version 28 (IBM, Chicago, IL, USA), was used for the statistical analysis.

The study was registered and approved by the local patient data safety council.

## Results

Seventy-nine patients were identified, and two patients were excluded as their diagnoses were later changed. Six patients were excluded because of missing US examination at baseline. The resulting 71 patients, 50 women (70.4%), with a confirmed diagnosis of GCA were included. The mean age was 69.7 years (SD: 7.2), range of 56–86 years. Apart from two patients (one Latin American and one from Thailand), all were of Caucasian origin (97.2%).

Characteristics of the cohort in accordance with the EULAR core criteria set are shown in Table 1.

**Table 1:** Characteristics of the GCA cohort of 71 patients in accordance with EULAR core data set.

		**All**	**c-GCA**	**LV-GCA**	**Mixed-GCA**	***P*-value**
**Total number of patients (%)**		***N** =* **71 (100%)**	***N** =* **22 (28.6%)**	***N** =* **12 (15.6%)**	***N** =* **33 (46.5%)**	
Demographics	Age	69.7 years (7.2)	69.8 (7.4)	70.3 years (9.2)	69.3 years (6.8)	0.915
	Female sex	50 (70.4%)	13 (61.9%)	9 (75.0%)	24 (72.7%)	0.635
	Weight	70.6 kg (14.3)	68.0 kg (14.6)	68.9 kg (11.5	73.4 kg (15.1)	0.381
	Height	168.2 cm (7.3)	168.2 cm (7.0)	167.3 cm (5.9)	168.9 cm (8.2)	0.826
	Smokers^*^	19 (26.8%)	9 (42.9%)	2 (16.7%)	7 (21.2%)	0.173
	Diagnostic delay (first symptom until diagnosis)	4.6 months (7.7)	2.6 months (3.5)	7.7 months (14.6)	5.1 months (5.3)	0.391
Cranial GCA-related signs and symptoms	Ocular symptoms	24 (33.8%)	10 (47.6%)	3 (25.0%)	9 (27.3%)	0.241
	Permanent/partial vision loss	2 (2.8%)	2 (9.5%)	0	0	0.110
	Headache	43 (60.6%)	16 (76.2%)	2 (16.7%)	21 (63.6%)	0.003
	Scalp tenderness	19 (26.8%)	6 (28.6%)	2 (16.7%)	8 (24.2%)	0.745
	Jaw claudication	22 (31.0%)	8 (38.1%)	2 (16.7%)	12 (36.4%)	0.396
	Cord-like thickening/ nodularity/ tenderness/ reduced/ pulse and/ or pulselessness	25 (35.2%)	10 (47.6%)	2 (16.7%)	11 (33.3%)	0.193
	Sonographic evidence of arteritis	65 (91.5%)	20 (95.2)	12 (100%)	33 (100%)	0.337
	Histological arteritis/biopsy^∧^	19 (26.8%)	5 (23.8%)	0	14 (42.4)%	
Constitutional	Fever/pyrexia symptoms	17 (23.9%)	4 (19.0%)	3 (25.0%)	10 (30.3%)	0.652
	Weight loss^¤^	20 (28.2%)	1 (4.8%)	5 (41.7%)	12 (36.4%)	0.018
	Night sweats^¤^	5 (7.0%)	2 (9.5%)	1 (8.3%)	2 (6.1%)	0.891
	Nausea or other constitutional symptoms^¤^		3 (14.3%)	0	10 (30.3%)	0.058
Laboratory	ESR	64.4 mm/t (31.6)	53.8 mm/t (22.1)	64.7 mm/t (41.0)	74.0 mm/t (30.9)	0.136
	CRP	76.26 mg/dl (82.4)	86.5 mg/dl (97.4)	71.3 mg/dl (59.2)	77.5 mg/dl (88.0)	0.917
	Hemoglobin	12 g/dl (1.7)	12.6 g/dl (1.7)	11.3 g/dl (1.7)	11.8 g/dl (1.6)	0.091
	Thrombocyte count¤	403.8 × 1000/μl (115.3)	411.7 × 1000/μl (89.6)	407.1 × 1000/μl (134.5)	386.9 × 1000/μl (121.6)	0.840
PMR	PMR	33 (46.5%)	11 (52.4%)	5 (41.7%)	16 (48.5%)	0.839
Arthralgia^¤^	Arthralgia^¤^	1 (1.4%)	0	0	1 (3.0%)	0.602
Dry cough^¤^	Dry cough^¤^	12 (16.9%)	1 (4.8%)	1 (8.3%)	9 (27.3%)	0.067
Large vessel/extra cranial involvement at diagnosis	Change in peripheral pulses or bruits over peripheral arteries	6 (8.5%)	2 (9.5%)	0	4 (12.1%)	0.456
	Blood pressure	145.5 (18.2)/80.2 (9.8) mmHg	147.6 (15.9)/80.8 (8.8) mmHg	141.4 (15.9)/76.8 (11.0) mmHg	146.9 (21.2)/80.7 (10.6) mmHg	0.672/0.539
	Dilatation/aneurysm	0	0	0	0	
	Inflammatory wall thickening (US, MR, CT)	65 (91.5%)	20 (95.2%)	12 (100.0%)	33 (100.0%)	0.337
	Stenosis	0	0	0	0	
	Carotidynia^¤^	3 (4.2%)	1 (4.8%)	1 (8.3%)	1 (3.0%)	0.752
Death	Death	0	0	0	0	
Cardiovascular events/conditions	Stroke or TIA (history of)	2 (2.8%)	1 (4.8%)	0	1 (3.0%)	0.602
	Myocardial infarction	1 (1.4%)	0	0	0	
	Arterial hypertension	33 (46.5%)	11 (52.4%)	4 (33.3%)	16 (48.5%)	0.556
Endocrine events and conditions	Diabetes	6 (8.5%)	2 (9.5%)	0	3 (9.1%)	0.542
	Osteoporosis	7 (9.9%)	2 (9.5%)	1 (8.3%)	4 (12.1%)	0.918
Infections	Active tuberculosis	0	0	0	0	
Malignancy	History of malignancy	7 (9.9%)	3 (14.3%)	2 (16.7%)	2 (6.1%)	0.476
Treatment	Prednisolone start dose	47.5 mg (12.8)	43.5 mg (13.5)	50.8 mg (17.7)	49.4 mg (10.3)	0.200
	Methylprednisolone	21 (29.6%)	8 (38.1%)	5 (41.7%)	7 (21.2%)	0.269
	Last dose after months of treatment^§^	33.5 months (21.8)	32.0 months (23.8)	26.9 months (7.1)	38.9 months (25.1)	0.319
	MTX	14 (19.7%)	2 (9.5%)	5 (41.7%)	6 (18.2%)	0.079
	Leflunomide	13 (18.3%)	4 (19.0%)	1 (8.3%)	6 (18.2%)	0.691
	Tocilizumab	11 (15.5%)	5 (23.8%)	1 (8.3%)	5 (15.2%)	0.490
	Gevokizumab	3 (4.2%)	0	0	3 (9.1%)	0.208
	Antiplatelet agents (ASA)	16 (22.5%)	4 (19.0%)	4 (33.3%)	6 (18.2%)	0.589
	Phenprocoumon	4 (5.6%)	0	1 (8.3%)	2 (6.1%)	
	Apixaban	3 (4.2%)	2 (9.5%)	0	1 (3.0%)	

Continuous variables are presented as mean with (SD) and categorical variables as frequency with (%). A comparison of groups was calculated between the three major GCA patterns as indicated. SD, standard deviation; ESR, erythrocyte sedimentation rate; CRP, C-reactive protein; PMR, polymyalgia rheumatic; US, ultrasound; CT, computed tomography; MR, magnetic resonance imaging; TIA, transient ischemic attack; MTX, methotrexate; ASA, acetylsalicylic acid. ^¤^Items are not part of the EULAR Core data set. ^*^Smoking status was recorded in 61 patients at diagnosis. ^∧^TAB was executed in 33 patients. Valid percent 46.5%. ^§^Ongoing treatment at the timepoint of data collection in 24 patients (33.8%).

The number of patients in our cohort fulfilling the original ACR 1990 classification criteria was 49 (69.0%), while 69 patients (97.2%) fulfilled the 2022 ACR/EULAR classification criteria. Table 2 shows the absolute number of patients fulfilling the separate criteria for the ACR 1990 classification criteria and the 2022 ACR/EULAR classification criteria. US was crucial for the classification of 27 patients (38.0%), while biopsy was crucial in one patient (1.4%).

**Table 2:** Comparison between the 1990 ACR criteria and the new 2022 EULAR/ACR criteria in our cohort of 77 patients diagnosed with GCA on a clinical basis.

**1990 ACR criteria**		**N (%)**	**2022 ACR/EULAR criteria**		**Points**	**N (%)**
Criterion 1	Age ≥ 50 years	71	Absolute requirement	Age ≥ 50 years at the time of diagnosis		71
Criterion 2	New onset of or new type of localized pain in the head	43 (60.6%)	Additional clinical criteria	Morning stiffness in shoulder/neck	+2	33 (46.5%)
				Sudden visual loss	+3	2 (2.8%)
				Jaw and tongue claudication	+2	22 (31.0%)
				New temporal headache	+2	43 (60.6%)
				Scalp tenderness	+2	19 (26.8%)
				Abnormal examination of the temporal artery	+2	25 (35.2%)
Criterion 3	Abnormal temporal artery palpation tenderness, decreased pulse	25 (35.2%)	Laboratory, imaging, and biopsy criteria	Maximum ESR ≥ 50 mm/h or maximum CRP ≥ 10 mg/liter	+3	66 (93.0%)
				Positive temporal artery biopsy or halo sign on temporal artery ultrasound	+5	66 (93.0%)
Criterion 4	ESR > 50 mm/h	56 (78.9%)		Bilateral axial involvement	+2	25 (35.2%)
Criterion 5	Abnormal artery biopsy	19/33 (57.6%)		FDG-PET activity throughout the aorta	+2	2/2
Number of patients fulfilling 1990 ACR criteria (%)		49 (69.0%)	Number of patients fulfilling 2022 ACR/EULAR criteria		69 (97.2%)	

In ACR 1990 criteria, ≥3 points are necessary to classify a patient as having GCA. In 2020 ACR/EULAR criteria, a score ≥6 is necessary to classify a patient as having GCA. ACR, American College of Rheumatology; EULAR, European League Against Rheumatism; ESR, erythrocyte sedimentation rate; CRP, C-reactive protein; FDG-PET, 18-F-FDG positron emission tomography with computed tomography localizer.

Detailed results for vasculitis distribution found by US examination are shown in Table 3.

**Table 3:** Distribution of the reported positive ultrasound vasculitis findings in the 71 patients receiving US at baseline.

	**Non-cranial large-vessel arteritis**		**Cranial arteritis of the temporal artery**		
	**Subclavian artery** ***N** =* **(%)**	**Axillary artery** ***N** =* **(%)**	**Common truncus** ***N** =* **(%)**	**Parietal branch** ***N** =* **(%)**	**Frontal branch** ***N** =* **(%)**
Right side	13 (16.8%)	36 (50.7%)	19 (26.8%)	20 (28.2%)	32 (45.1%)
Left side	13 (16.8%)	27 (38.0%)	18 (25.4%)	18 (25.4%)	32 (45.1%)
Total	15 (19.5%)	38 (53.5%)	22 (31.0%)	20 (28.2%)	40 (56.3%)
Total	41 (57.7%)		45 (63.4%)		

Mixed-GCA was observed in 33 patients (46.5%) patients, c-GCA in 22 (28.6%) patients, and LV-GCA in 12 (15.6%) patients. Nine patients had a positive finding at just one site. Five patients had isolated unilateral subclavian vasculitis, and two patients had isolated unilateral frontal artery and superficial artery involvement each.

In five patients (7.0%), the diagnosis was based on clinical grounds only without evidence of vasculitis in ultrasound (all five patients), biopsy (four patients), or magnetic resonance (one patient). The ACR 1990 classification criteria were fulfilled by 14 patients (66.7%) in the c-GCA group, 3 patients (25%) in the LV-GCA group, and 29 patients (87.9%) in the mixed-GCA group. The 2022 ACR/EULAR classification criteria were fulfilled in all patients with positive ultrasound findings, irrespective of the subtype but only in three of the five patients (60%) without evidence of vasculitis in the US examination.

Three ischemic events in two patients were observed. One patient who already received treatment with aspirin for concomitant diagnosis developed a posterior stroke as well as an anterior ischemic optic neuropathy, and another patient without aspirin or oral anticoagulation treatment developed an anterior optic neuropathy. Of the seven patients on oral anticoagulation treatment, none developed ischemic complications. The paucity of ischemic events precluded a further associative analysis.

Weight loss was significantly more frequent in patients with large-vessel non-cranial involvement (*p* = 0.018), but between mixed-GCA and LV-GCA, no significant difference was found. Headache was significantly more frequent in cranial vasculitis in c-GCA and mixed-GCA compared with LV-GCA (*p* = 0.003). No significant differences between GCA patterns could be demonstrated for other characteristics from the EULAR core data set nor arthralgia, dry cough, carotidynia, night sweats, and other constitutional symptoms. The three events of new vision loss were seen in two c-GCA patients.

## Discussion

In this study, we present all patients in Agder County who were diagnosed with GCA in the given period and underwent expert ultrasound to characterize the extent of the vasculitis. However, this study comes with relevant shortcomings. Among others, they encompass, that some parts of the vasculature deemed relevant, such as the vertebral-, occipital-, and facial arteries but also the aorta, were inconsequently or never assessed (2, 6, 27). US follow-up data and IMT were not documented (28). Incomplete data were collected in the follow-up regarding medication dose, steroid tapering, steroid toxicity, and relapse. As no data on patients, in which a GCA diagnosis was rejected in the FTC was collected, no conclusion on the performance of the two criteria sets could be made.

Mixed-GCA was the most common form in our cohort, confirming recent findings (6, 9, 29–32). Mixed-GCA was observed in 33 patients (46.5%), c-GCA in 22 patients (28.6%), and LV-GCA in 12 patients (15.6%). Our data highlight the importance of an extended US examination of cranial and non-cranial large arteries for diagnosing GCA in daily clinical care, comparable to other recent literature (6, 7, 9). The US data demonstrated the widespread nature of arterial inflammation in GCA that rarely involves only one site. However, the relatively lower numbers of large-vessel vasculitis compared with other studies may be a consequence of an often-limited US examination executed in this cohort, only occasionally encompassing subclavian, carotid, aortic, vertebral, facial, or occipital arteries (6). Furthermore, the training and experience of sonographers varied as well as US machines. This may also explain why five (7.0%) patients showed no objective vasculitis in the US examination and nine patients were identified with just one single involved vascular site. An US was executed after a maximum of three oral doses of prednisolone. Even though some vasculitic changes, especially in the cranial vasculature, may have vanished by then, in our cohort, LV-GCA showed a trend toward a longer diagnostic delay that did not reach significance (14, 33). Six patients were excluded due to a missing ultrasound at baseline. Only two of these patients underwent TAB and PET. Both modalities showed positive findings in these two patients. The remaining four patients were solely diagnosed by TAB without further assessment of possible large-vessel vasculitis.

Headache was significantly associated with cranial vasculitis. However, no significant difference between c-GCA and mixed-GCA could be demonstrated (7, 17, 18, 29). Weight loss was significantly associated with vasculitis in large non-cranial vessels, but no further significant difference between LV-GCA and mixed-GCA could be shown. In contrast to other studies, neither age, sex, treatment length, nor any laboratory markers differed significantly between the three patterns (14, 29, 33).

The 2022 ACR/EULAR classification criteria allocated a much higher proportion of our US-based FTC cohort as having GCA than the 1990 ACR classification criteria. This is in accordance with other recent cohort studies (16, 34, 35). This was especially true for the LV-GCA subgroup where only 25% of the patients would have been classified as having GCA using the 1990 ACR classification criteria, while all patients fulfilled the 2022 ACR/EULAR classification criteria. As previously demonstrated in FTCs, ischemic complications were few as only two patients (2.8%), both with c-GCA, developed three ischemic events (17, 18). However, diagnostic delay based on retrospective first symptom occurrence to the specialist investigation was 4.6 (SD: 7.7) months despite an established FTC that is set up to see patients on the next working day. This potentially mirrors the unspecific nature of symptoms that both the patient and the primary health service are confronted with in GCA patients. Treatment length, indicated by the last corticosteroid dose, reflected on the one hand the relapsing nature of GCA and on the other hand the need for steroid-sparing strategies. In our small cohort, GCA subgroups by US stratification alone were associated with some clinical features. However, this approach was insufficient to predict the duration of the treatment, indicating the need for better risk stratification using improved imaging parameters or scores as well as laboratory markers (36, 37).

## Conclusion

Our study confirms that GCA is a multisite vasculitis with distinct clinical features depending on the involved vessels. This should be considered in any workup procedure. 2022 ACR/EULAR classification criteria allocated a much higher percentage of our GCA cohort (97.2%) as having GCA compared with the 1990 ACR classification criteria (69.0%) and reflected the clinical practice in our FTC better.

## Data availability statement

The raw data supporting the conclusions of this article will be made available by the authors, without undue reservation.

## Ethics statement

The studies involving humans were approved by local patient data safety council, Sørlandets Sykehus, Kristiansand, Norway. The studies were conducted in accordance with the local legislation and institutional requirements. Written informed consent for participation was not required from the participants or the participants' legal guardians/next of kin in accordance with the national legislation and institutional requirements.

## Author contributions

PA: Conceptualization, Investigation, Methodology, Project administration, Writing—original draft, Writing—review & editing. AD: Investigation, Supervision, Writing—review & editing. GM: Supervision, Writing—review & editing. GH: Conceptualization, Funding acquisition, Investigation, Methodology, Project administration, Resources, Supervision, Writing—review & editing.
